# Anti-tumor activity of a recombinant soluble Fzd7 decoy receptor in human gastric and colon cancer cells

**DOI:** 10.22038/IJBMS.2022.61908.13700

**Published:** 2022-02

**Authors:** Nasim Hafezi, Reza Valadan, Omran-Hossein Asgarian, Abolghasem Ajami

**Affiliations:** 1 Department of Immunology, School of Medicine, Mazandaran University of Medical Sciences, Sari, Iran; 2 Molecular and Cell Biology Research Center, Faculty of Medicine, Mazandaran University of Medical Sciences, Sari, Iran; 3 Gastrointestinal Cancer Research Center, Non‐Communicable Diseases Institute, Mazandaran University of Medical Sciences, Sari, Iran; 4 Department of Infectious Diseases, Antimicrobial Resistance Research Center, Mazandaran University of Medical Sciences, Sari, Iran

**Keywords:** Anti-tumor activity, Colon cancer, Decoy receptor, Gastric cancer, Recombinant protein, Soluble Fzd7

## Abstract

**Objective(s):**

Frizzled-7, the most common receptor of the Wnt signaling pathway, was significantly over-expressed in gastric (GC) and colorectal (CRC) cancers and stimulated tumorigenesis. The extracellular domain of Fzd7 (sFzd7) as a decoy receptor, could competitively bound with ligands and antagonize the interaction between Fzd7 receptors and Wnt ligands.

**Materials and Methods:**

We expressed and purified the extracellular region of Fzd7 including cysteine-rich domain (33 aa–185 aa) from *Escherichia coli* by chromatography. The effect of sFzd7 was evaluated on AGS gastric and SW480 colon cancer cell lines expressing high levels of Fzd7 receptor. Accordingly, cell viability and apoptosis were measured using MTT and flow cytometry assays, respectively. Real-Time PCR determined the relative expression of the β-catenin and cyclin-D1 genes.

**Results:**

After three days of treatment with sFzd7, the viability of AGS and SW480 cell lines was decreased in a dose-dependent manner. In addition, sFzd7 at concentrations of 10 and 20 ug/ml increased the rate of apoptosis. Especially at the concentration of 20 ug/ml, the apoptosis rate was remarkably high in AGS (P-value= 0.003) and SW480 cells (*P*-value= 0.0007). Finally, the expressions of β-catenin (*P*-value= 0.01) and cyclin-D1 (*P*-value= 0.02) were obviously decreased in SW480 cells. The same results were obtained in AGS cells, although not statistically significant.

**Conclusion:**

sFzd7 decoy receptor inhibits tumor cell progression by attenuating the Wnt pathway through inhibiting Fzd7 receptors and Wnt ligand interaction. Hence, sFzd7 can be proposed as a candidate therapy for GC and CRC cells with high levels of Fzd7 expression.

## Introduction

Gastrointestinal (GI) cancers are responsible for 26% of global cancer incidence and 35% of all cancer-related deaths (1). Colorectal (CRC) and gastric (GC) cancers pose the most frequently GI cancers with respectively,1.8 and 1.0 million new cases each year (2). In both of these cancers, diagnosis happens frequently in the late stages of cancer along with a poor response to treatment (3). 

Past evidence indicates that overactivation of the Wnt signaling pathway has been implicated in the pathological process of GI cancers including metastasis, chemoresistance, and tumor relapse by maintenance of cancer stem cells (4, 5). The Wnt pathway is initiated by the formation of a complex between Wnt ligand, Fzd receptors, and some co-receptors, resulting in the activation of distinct pathways, including coactivator β-catenin dependent pathway (canonical) and β-catenin-independent pathways (noncanonical) (6).

Frizzled-7 (Fzd7) is a member of the seven-transmembrane family of Fzd receptors that is composed of an extracellular cysteine-rich domain (CRD) along with transmembrane segments and an intracellular domain. CRD domain is the interaction site with Wnt ligands which is the first step in the initiation of downstream signaling pathways (7).

Up-regulation of Fzd7 is implicated in several human cancers caused by activation of Wnt pathways accompanied by an increase in cell proliferation, invasion, metastasis, and recurrence (8). The overexpression of Fzd7 has been shown in both GC and CRC cells and is associated with tumor invasion, lymphatic and organ metastasis, and poor patient survival (9-11). Furthermore, the knockdown of the Fzd7 gene by small interference RNAs (siRNAs) effectively decreased the tumorigenesis features of GC and CRC cells (12, 13). Hence, targeted inhibition of Fzd7 could be a promising approach for the treatment of GI cancers. 

The CRD domain of Fzd receptors has an inevitable role in beginning and stimulating of the Wnt pathway. A soluble Fzd7 CRD as an Fzd decoy receptor, could modulate the up-regulation of Wnt signaling via binding and blocking Wnt ligands. For this reason, targeting Fzd7 CRD may be an effective therapeutic approach for cancers with overactive Wnt signaling pathways. In this study, we aim to produce the recombinant soluble Fzd7 (sFzd7) consisting of the extracellular region encompassing the CRD domain. Subsequently, we assessed the anti-tumor activity of sFzd7 in gastric and colon cancer cell lines. 

## Materials and Methods

***Cell lines and primary cell cultures***

The human gastric cancer cell line AGS (ECACC 89090402) and human colon cancer SW480 (donated by Cancer Research Center of Shiraz Medical University (Shiraz, Iran)) were maintained in RPMI medium (Gibco, USA) supplemented with 10% (v/v) FBS (Gibco, NZ), 100 μg/ml penicillin and 100 μg/ml streptomycin. Cells were maintained at 37 °C in a humidified atmosphere with 5% CO_2_.

***Construction and purification of recombinant rhFzd7***

The sequence of homo Fzd7 extracellular domain (sFzd7) from nucleotide 97-555 was proliferated from Complementary DNA (cDNA) of human peripheral blood mononuclear cells (PBMCs) using polymerase chain reaction (PCR). *BamHI* and *EcoRI* cleavage sites and also the TGA last codon was added to the sequence of sFzd7 by PCR with the following primers: upstream (GGA TCC CAG CCG TAC CAC GGA GAG) and downstream (G AAT TCT CAC AGG TAG GGC GCG GTA GGG TAG). The PCR reaction was conducted using an Applied Biosystems (ABI) system under the following conditions: heat activation of the polymerase for 2 min at 94 °C, followed by 30 cycles of 94 °C for 30 sec, 62 °C for 30 sec, and 72 °C for 45 sec; with a final extension at 72 °C for 5 min. The PCR amplified product was run with an agarose gel electrophoresis and the considered band isolated by gel extraction. Then, the product was cloned into the pTG19-T cloning vector and transformed into *Escherichia coli* TOP-10 strain. The sequence of sFzd7 was confirmed by Bioneer Co. Subsequently, pET-28a (+) expression vector containing His-tag at the N- region of the multi-cloning site, was used to construct the His-tagged sFzd7 protein. The sequence of sFzd was sub-cloned into the expression vector pET-28a (+) (Novagen, Germany), by digestion with *BamHI* and *EcoRI* restriction endonuclease. The recombinant vector pET-28a (+)-sFzd7 was introduced into *E. coli* SHuffle™ T7 strain. Recombinant sFzd7 protein was produced with IPTG induction (0.5 mM, 37 °C, 220 rpm, 5 hr) and purified from the insoluble fraction of the bacterial lysate by using 8 M urea, 2ME 10 mM, and triton x100 (PH 8). Purification was done with the Ni-NTA affinity column by using AKTA purifier chromatography device and eluted by elution buffer containing 250 mM imidazole (14**)**. The concentration of final His-rsFzd7 recombinant protein was determined by Bradford protein assay (Bio basic, Ontario, Canada), and purification was confirmed by SDS-PAGE and immunoblotting using Goat anti-Fzd7 antibody (R&D, cat.no. AF198-SP) and HRP-conjugated Sheep anti-goat IgG (R&D, cat.no. ab6741).

***Validation of Fzd7 receptors expression on the surface of AGS and SW480 cell lines by flow cytometry***

AGS and SW480 cell lines at the density of 5 × 10^5^ per sample were washed and suspended in PBS containing 0.5% BSA. The cells were incubated with 6 µg/ml Rabbit anti-mouse Fzd7 (Abcam, cat.no. ab64636) at 4 °C for 30 min. Then, cells were incubated with FITC-conjugated anti Rabbit IgG antibody (Abcam, cat.no. ab6791) at 4 ℃ for 30 min followed by being washed twice with PBS. The flow cytometric assay was performed using a Partec flow cytometer (Partec PAS, Germany) and the data were analyzed by FlowJo software 7.6.1 (Tree Star, Inc., San Carlos, CA, USA).

***Cell viability assay***

AGS and SW480 cell lines were seeded in 96-well plates at 5 × 10^3^ and 10 × 10^3^ cells/well, respectively. The cells were maintained overnight at 37 °C and then incubated with different concentrations of sFzd7 in triplicate from 1.56-50 µg/ml at 37 °C for 72 hr. All assays were performed in triplicate. The cells treated with vehicle buffer served as control. After incubation time, MTT solution (at a final concentration of 0.5 mg/ml) was added to each well at 37 °C for 4 hr. Then, the culture supernatant was removed and the resulting formazan was solubilized with DMSO. Finally, for the determination of absorbance density values, the plates were read by an enzyme-linked immunosorbent assay (ELISA) reader at 570 nm, and the cell viability rate was expressed as percentage of the vehicle control (100%). 

***Apoptosis assays***

40 × 10^3^ AGS and 80 × 10^3^ SW480 cells were seeded in 12-well plates and maintained overnight at 37 °C. The cells were treated with rsFzd7 in triplicate at 10 and 20 µg/ml and then stained with Annexin V (AV)-FITC and propidium iodide (PI) to distinguish early apoptotic (AV+/PI−), late apoptotic (AV+/PI+), and necrotic (AV−/PI+) populations using manufacturer’s protocol of FITC Annexin V Apoptosis Detection Kit (IQ Products, USA). Finally, the data of apoptosis were detected in a Partec flow cytometer (Partec PAS, Germany).

***Real-time PCR (RT-PCR) assay***

The effect of sFzd7 on the β-catenin and cyclin D1 RNA expression was verified by quantitative real-time PCR (qRT-PCR). AGS and SW480 cells were treated in triplicate with sFzd7 (15 µg/ml) for 72 hr. Subsequently, the total RNA was extracted by TRIzol reagent and purified with the RNeasy Mini Kit (QIAGEN, CA, USA). 500 ng/µl RNA was reverse-transcribed into cDNA using a cDNA synthesized kit (Fermentas, USA). RT-PCR was performed using the SYBR master mix (Amplicon) with the following primers in an ABI plus one system. β-catenin: upstream primers: 5’- TGCTATCTGTCTGCTCTAGTA -3’, downstream primers: 5’ CTTCCATCCCTTCCTGTTTAG -3’, cyclin D1: upstream primers: 5’- CCTCGGTGTCCTACTTCA -3’, downstream primers: 5’ CTCCTCGCACTTCTGTTC -3’) and GAPDH: upstream primers: 5’- GGTGGTCTCCTCTGACTTCAACA -3’, downstream primers: 5’ GTTGCTGTAGCCAAATTCGTTGT -3. After an initial denaturation for 3 min at 95 °C, qRT-PCR was followed by 40 cycles at 95 °C for 30 sec, at 60 °C for 30 sec, and 72 °C for 30 sec. The relative mRNA expression was calculated by the comparative CT (2^−ΔΔCT^) method.

***Statistical analysis***

Statistical analysis was performed using GraphPad Prism^TM^ software version 6 (GraphPad Software Inc., CA, USA). Values were expressed as mean ± standard error (mean ± SEM).

The Findings were considered significant when *P*-values were <0.05. 

**Figure 1:**
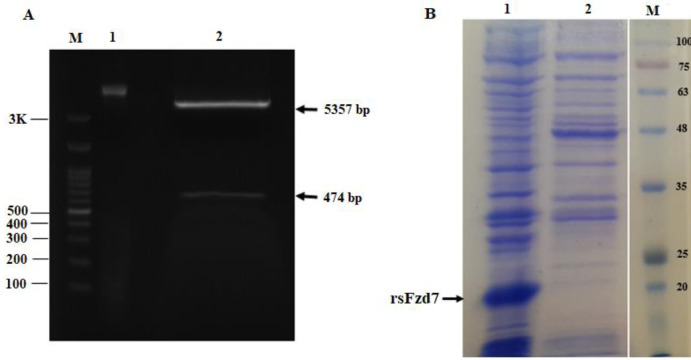
Cloning of the extracellular region of Fzd7 (sFzd7) from 33 aa- 185 aa in pET28a (+) expression vector and expression in *Escherichia coli* SHuffle™ T7 strain. (A) recombinant pET28a (+)-Fzd7 vector was confirmed by digestion with BamHI and EcoRI restriction enzymes: M= DNA marker, Lane 1 = undigested vector, lane 2 = digested vector. (B) SDS-PAGE of sFzd7 expressed in SHuffle™T7 strain: M= protein marker, lane 1 = un-induced bacteria, lane 2 = induced bacteria with IPTG

**Figure 2:**
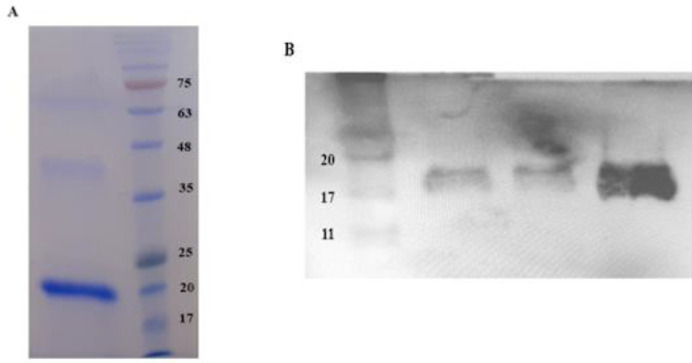
Purification of sFzd7 by Ni-NTA affinity chromatography and identification through (A) SDS-PAGE and (B) Western blotting. 20 kDa sFzd7 protein was clearly detected

**Figure 3:**
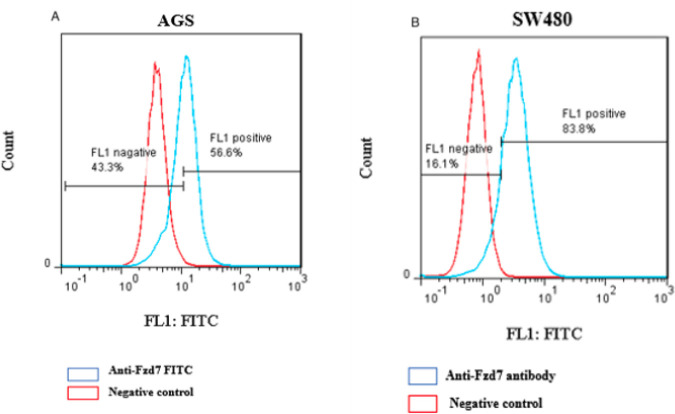
Anti-human Fzd7 antibody exhibited relatively high binding rate with AGS and SW480 cells at 56.6% and 83.8%, respectively

**Figure 4:**
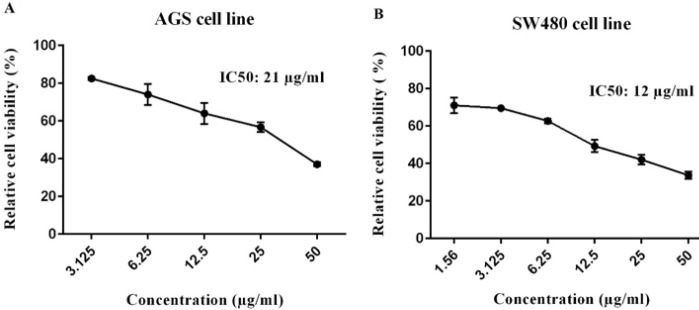
AGS and SW480 cells were treated with sFzd7 at different concentrations (1.56, 3.125, 6.25, 12.5, 25, and 50 µg/ml) for 72 hr, and cell viability was detected by MTT assay. (Data were presented as mean ± SE). IC_50_ value of sFzd7 was obtained (A) 21 µg/ml for AGS and (B) 12 µg/ml for SW480 cells

**Figure 5:**
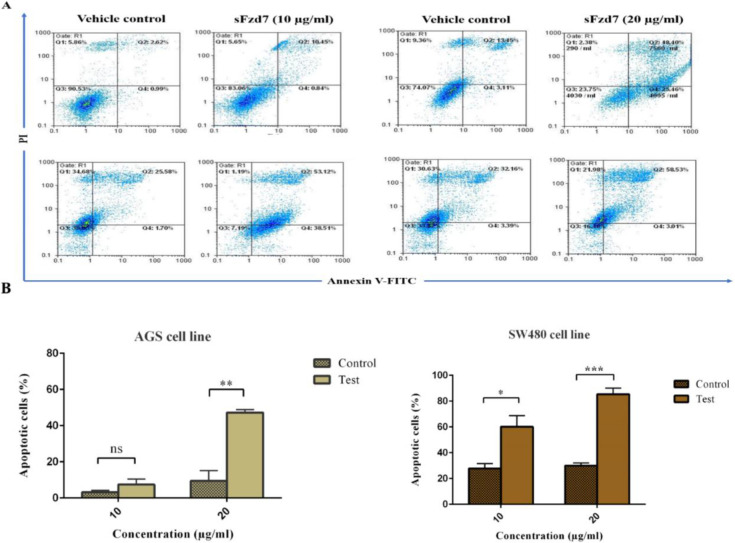
(A) In the top and bottom row images are shown the apoptosis rate of AGS and SW480 cells cultured in the presence or absence (vehicle control) of rsFzd7 at 10 and 20 µg/ml, respectively. (B) Percentage of apoptotic AGS and SW480 cells is represented. Results are expressed as means ± SE of three independent experiments. Unpaired t-test was used for analyses (*P<0.05, **P<0.01, ***P<0.001)

**Figure 6:**
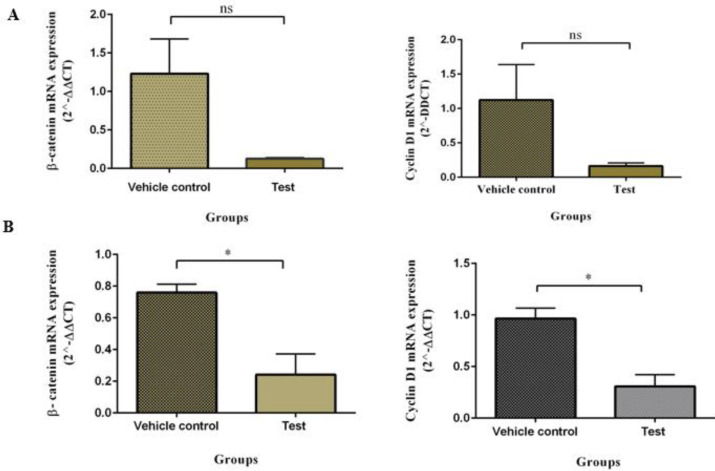
In the top and bottom rows, the level of β-catenin and cyclin D1 mRNA expression compared with vehicle control is represented using quantitative real-time PCR, respectively, in AGS and SW480 cells. The data were reported as mean±SE of three independent experiments. Unpaired t-test was used for analyses (*P<0.05)

## Results

***Expression and identification of sFzd7***

The pET28a (+)-sFzd7 construct was confirmed by digestion with *BamHI* and *EcoRI* restriction enzymes and the presence of 474 bp band was associated with sFzd7 (Figure 1A). Furthermore, the accuracy of the construct was verified by DNA sequencing (data not shown). According to the presence of five disulfide bonds in the extracellular cysteine-rich domain (CRD) of Fzd7, we used the* E. coli* SHuffle^™^ T7 strain that is a preferred choice for the expression of proteins with multiple disulfide bonds. The recombinant pET28a (+)-sFzd7 plasmid was transformed into SHuffle^™^ T7 strain and the expression was induced at 0.5mM IPTG, for 5 hr at 37 °C. As shown in Figure 1B, the sFzd7 protein with a molecular weight of 20kDa was clearly observed. All recombinant expressed protein was observed in the insoluble fraction. Thus, the solubilization of sFzd7 was tried by changing the induction temperatures, incubation time, shaking speed, and concentration of IPTG. But, in the end, no protein band was obtained from the supernatant fraction of cell lysate. The protein was purified from the insoluble fraction and lysed by a buffer containing 8 M urea. The protein was refolded by urea depletion and eluted by elution buffer with 250 mM imidazole (PH 8). SDS-PAGE analysis confirmed the purity of the isolated protein (Figure 2A). Western blot analysis utilizing anti-human Fzd7 antibody (Figure 2B) indicated the successful expression of rhFzd7 and the apparent molecular weight of rhFzd7.

***sFzd7 inhibited proliferation of AGS and SW480 cells***

FCM analysis showed that anti-human Fzd7 antibodies exhibited a relatively high binding rate with AGS and SW480 cells. The frequencies of the Fzd7 receptor were 56.6% and 83.8% for AGS and SW480 cells, respectively (Figure 3) which indicates the highly-expressed Fzd7 on AGS and SW480 cells.

MTT assay was carried out to assess the inhibitory effect of the sFzd7 decoy receptor on cell viability of AGS and SW5480 cell lines. 

The results showed that sFzd7 treatment effectively reduced the cell growth of AGS and SW48 cell lines in a dose-dependent manner with 50% growth inhibition (IC50) value 21 and 12 µg/ml, respectively (Figure 4). 

***sFzd7 induced apoptosis in AGS and SW480 cell lines***

Apoptosis assay was performed to identify the ability of sFzd7 to induce apoptosis in AGS and SW480 cell lines. Apoptosis was evaluated in AGS and SW480 treated with 10 and 20 µg/ml sFzd7. Cells treated with vehicle alone served as the control. The apoptosis rate of cells was assayed with FITC- conjugated Annexin V/PI staining using flow cytometry.

As demonstrated in Figures 5 A and B, 10 µg/ml sFzd7 induced 10.45% cell apoptosis of AGS cells (*P*-value= 0.08); as for SW480 cells, sFzd7 induced 53.12% cell apoptosis compared with the control (*P*-value= 0.04). At 20 µg/ml sFzd7 induced 48.4% cell apoptosis (*P*-value= 0.003) of AGS; as for SW480 cells, sFzd7 induced 83.36% cell apoptosis (*P*-value= 0.0007).

***sFzd7 reduced the expression of β-catenin and cyclin D1 in AGS and SW480 cell lines***

We used the quantitative RT-PCR assay to analyze the expression status of β-catenin and cyclin D1 in AGS and SW480 cell lines treated by sFzd7. As shown in Figure 6, the mRNA levels of β-catenin and cyclin D1 were down-regulated in AGS cells. However, this difference was not significant (*P*-value= 0.07 and *P*-value= 0.08, respectively). In SW480 cells a significantly decreased level of β-catenin and cyclin D1 appeared (*P*-value= 0.01 and *P*-value= 0.02, respectively). 

## Discussion

In this study, we indicated the anti-tumor activity of an sFzd7 decoy receptor in two cancers, GC and CRC. Fzd7 is a key receptor of the Wnt pathway, that is up-regulated in GC and CRC cells and associated with tumor invasion, metastasis, and cancer stem cell formation (15, 16). Accordingly, targeted inhibition of Fzd7 exhibits a promising therapeutic option for GC and CRC cancers.

Decoy receptors are the ectodomain of cell surface receptors with ligand binding ability and are proposed as a therapeutic strategy for targeting oncogenic receptors via blocking the ligand-receptor interactions (17, 18). So far, a soluble vascular endothelial growth factor (VEGF) decoy receptor entitled aflibercept has been approved for cancer therapy (19).

In the present study, we expressed and purified the recombinant soluble Fzd7 decoy receptor composed of an extracellular region of Fzd7 (sFzd7). The binding of the sFzd7 receptor to Wnt ligands could obstruct the interaction between Wnt and membrane Fzd receptors. Hence, Fzd7 blockade may lead to the suppression of tumor cell growth by inhibiting the Wnt signaling pathway. We first produced the recombinant sFzd7 in a prokaryotic system. Due to the presence of five disulfide bonds in the Fzd7 extracellular CRD domain, we used the SHuffle^TM^ T7 strain which is engineered to express proteins with disulfide bonds and yield soluble proteins (20). However, the recombinant sFzd7 was found just in the insoluble inclusion bodies, so the pellet containing protein was dissolved in 8 M urea and then refolded through the elimination of urea. 

In the next step, the functionality of sFzd7 was assessed on AGS human GC and SW480 CRC cell lines with high expression of the Fzd7 receptor. The sFzd7 dose-dependently inhibited the cell viability in both cell lines. Moreover, we found that sFzd7 treatment significantly elevated the apoptosis rate in AGS and SW480 cells. Furthermore, the expression levels of β-catenin and cyclin D1 genes were decreased in both treated cell lines. The decreasing level of β-catenin and cyclin D1 could explain that sFzd7 regulates tumor cell growth in the β-catenin-dependent pathway. Previous studies indicated that β-catenin plays a critical role in the process of tumorigenesis by overexpressing the target genes of the Wnt pathway such as cyclin D1 (21). Cyclin D1 plays an important role in cell division and is associated with the progression of GC and CRC (22, 23).

The biotherapy effect of the soluble Fzd receptors against tumors was first presented by DeAlmeida *et al*. who demonstrated that soluble Fzd8 CRD domain could serve as a potential anti-tumor agent in teratocarcinoma cells (24). Further, the efficacy of the soluble Fzd7 has been taken into consideration and assessed thus far in two types of cancer, hepatocellular carcinoma (HCC) and triple breast cancer cells (TNBC) (14, 25). These studies in consistence with our data reported that the soluble Fzd7 CRD domain exerts growth suppressor effects along with reduced expression of cyclin D1, c-Myc, and survivin. Furthermore, soluble Fzd7 augmented the chemotherapeutic agents’ effect on inhibiting tumor growth. These findings provide strong evidence for the influential role of the sFzd7 decoy receptors in blocking the Wnt/ β-catenin pathway activity by competitive binding of the Fzd7 CRD domain with Wnt ligands. 

## Conclusion

The present study revealed that the recombinant sFzd7 protein by acting as a decoy receptor can inhibit the growth of tumor cells and block the Wnt signaling pathway in GC and CRC cells. Thus, we propose that sFzd7 can be used as a therapeutic choice for GC and CRC. But, the clinical applicability of sFzd7 should be further determined in* in vivo *assays and in combination with chemotherapeutic agents.

## Authors’ Contributions

NH Performed the experiments and wrote the manuscript. RV helped in protein purification. HA-O Helped design the experiments. AA Designed the study, edited the manuscript, and approved the final draft. All authors read and approved the final manuscript. 

## Conflicts of Interest

The authors declare that they have no conflicts of interest.
